# Hyperbaric Oxygen Therapy Is an Effective Adjunctive Therapy to Manage Treatment-Resistant Venous Leg Ulcers

**DOI:** 10.3400/avd.cr.21-00030

**Published:** 2021-09-25

**Authors:** Kotaro Suehiro, Motoki Fujita, Noriyasu Morikage, Takasuke Harada, Makoto Samura, Ryo Suzuki, Hiroshi Kurazumi, Ryosuke Tsuruta, Kimikazu Hamano

**Affiliations:** 1 Division of Vascular Surgery, Department of Surgery and Clinical Science, Yamaguchi University Graduate School of Medicine, Ube, Yamaguchi, Japan; 2 Acute and General Medicine, Yamaguchi University Graduate School of Medicine, Ube, Yamaguchi, Japan

**Keywords:** hyperbaric oxygen therapy, venous leg ulcer, chronic venous insufficiency

## Abstract

We report five cases of venous leg ulcers (VLU) that were resistant to conservative therapy for 22–119 months and were eventually healed via hyperbaric oxygen therapy (HBOT). In one patient, VLU recurred four times and was managed using HBOT, each time. The VLU sizes ranged from 18 to 68 cm^2^ before HBOT. HBOT was administered at 2.0 atmospheres absolute with 100% oxygen for 60 min per session, five sessions a week during hospitalization. All VLUs healed after 17–66 sessions of HBOT.

## Introduction

Most venous leg ulcers (VLU) heal with compression therapy as recommended by the guidelines.^1,2)^ However, unhealed VLU can persist for years or increase in size despite appropriate treatment, in which adjunctive therapies will be inevitably required. Among the popular adjunctive therapies for VLU,^3)^ human skin substitutes are not available in Japan. Negative pressure wound therapy (NPWT)±skin graft may be another option; however, the available duration for NPWT covered by the National Health Insurance is not long enough and a skin graft may not be appropriate for patients with multiple recurrences. In 2015, we treated a patient with nonhealing mixed, i.e., ischemic and venous, ulcer using NPWT. Unfortunately, his wound was complicated by a serious infection caused by anaerobic bacteria. To treat this infection as well as his ulcer, we used hyperbaric oxygen therapy (HBOT). Besides settlement of infection, we noticed an unexpected and significant improvement of his ulcer. Thereafter, we started to use HBOT for the treatment of VLUs. In this report, we present treatment-resistant VLUs in five patients, who were successfully managed with HBOT.

## Case Report

This case series was approved by the Institutional Review Board of Yamaguchi University Hospital (Center for Clinical Research, Ube, Yamaguchi, Japan; 9999-006-[2]). Between April 2016 and November 2020, 53 patients with VLU were referred to and treated in our outpatient clinic by compression therapy using bandages. We previously reported that the 6 and 12 month healing rates of this conservative treatment were 67% and 86%, respectively.^4)^ Among them, the VLUs in five patients either did not shrink or further increased in size over 6 months of treatment, i.e., treatment-resistant, and these patients were subsequently treated using HBOT. **Table 1** summarizes the patient characteristics. VLU in case 1 recurred four times and was treated with HBOT each time. On their initial visits, the trace of the great saphenous vein (GSV) stripping but no recurrence was confirmed in cases 1 and 2 through a duplex venous scan. In cases 3, 4, and 5, axial reflux in GSV was confirmed. No deep vein occlusion and insufficiency were found in any of the cases. Thus, the initial causes of their VLUs were considered to be the GSV reflux and additional patient-related risk factors (obesity, fixed ankle, and prolonged standing) listed in **Table 1**. GSV reflux in cases 3, 4, and 5 were treated in our clinic within 2 weeks from their initial visits. Accordingly, the possible cause of resistance to the conservative treatment was considered to be patient-related risk factors. These factors can cause VLU even without venous insufficiency confirmed via duplex venous scan,^5)^ and since these factors are very difficult to fix, they seemed good reasons for treatment-resistant or recurrent VLUs.

**Table 1:** Table 1 Patient characteristics and treatment outcomes

Case	Age (years)	Sex	Side	BMI >30 kg/m^2^	Occupation standing >8 h	Fixed ankle	VLU duration before HBOT (months)	Initial VLU size (cm^2^)	HBOT sessions required
1	37	Male	Left	+	+	—	60	68	28
Recurrence 1	39	Male	Left	+	+	—	6	96	25
Recurrence 2	40	Male	Left	+	+	—	7	122	39
Recurrence 3	40	Male	Left	+	+	—	6	90	66
Recurrence 4	41	Male	Left	—	+	—	6	42	27
2	84	Female	Right	—	—	+	60	39	42
3	67	Female	Left	—	+	—	22	18	23
4	83	Female	Left	—	—	+	54	20	63
5	75	Female	Left	—	—	+	119	48	17

BMI: body mass index; VLU: venous leg ulcer; HBOT: hyperbaric oxygen therapy

**Figure 1:**
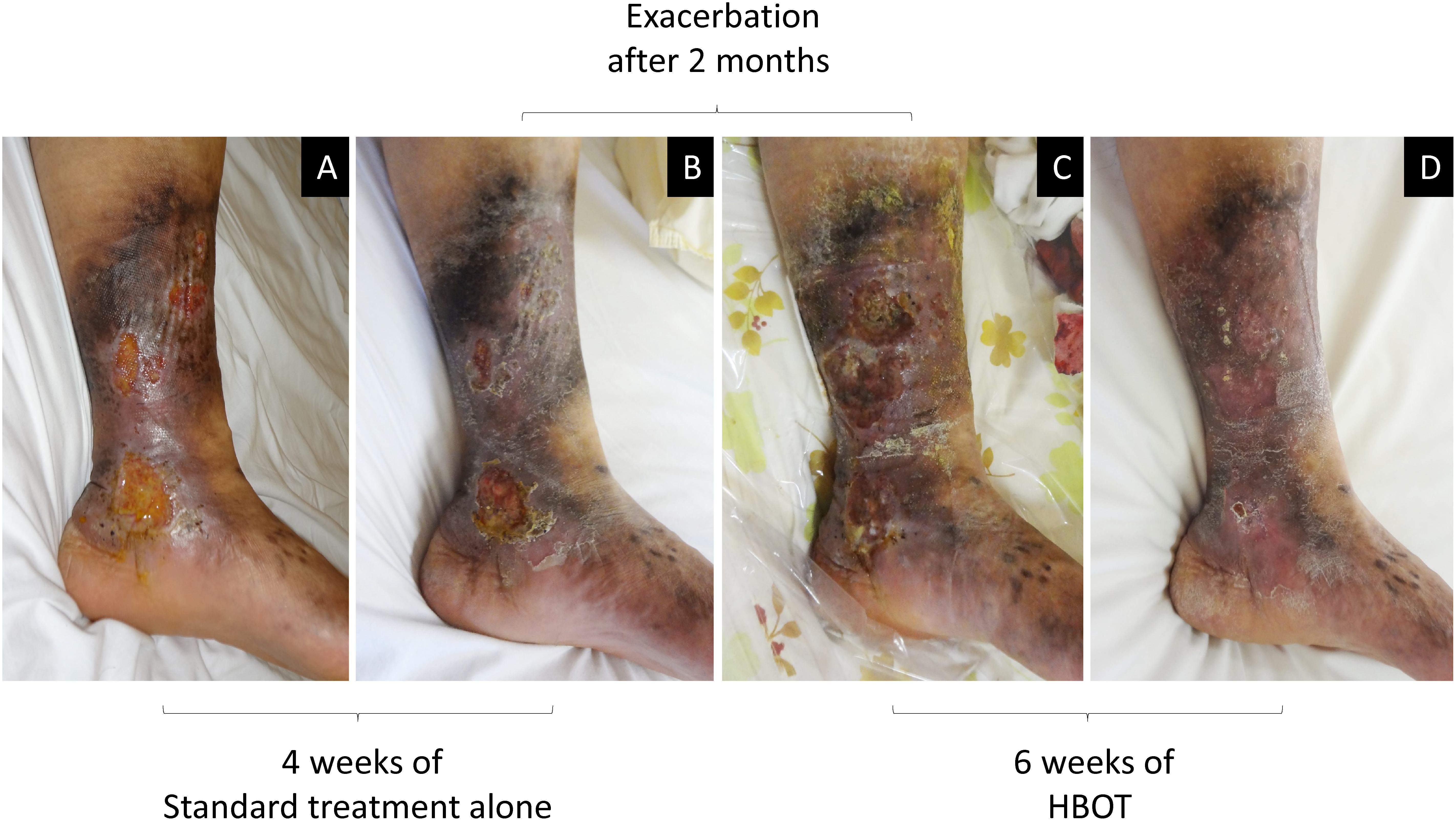
Fig. 1 Venous leg ulcer (VLU) in case 1 before and following first-time hyperbaric oxygen therapy (HBOT). A 37-year-old man, who worked in a soba restaurant standing for 11 h every day, had VLU for nearly 5 years (**A**). He was admitted for 4 weeks, and a standard treatment was administered, which improved the condition slightly (**B**). Two months later, he returned with seriously exacerbated VLU (**C**). After readmission, 28 sessions of HBOT (6 weeks) were administered, and the VLU eventually healed (**D**).

All patients were admitted to Yamaguchi University Hospital and HBOT was performed using 100% oxygen for 60 min at 2.0 atmosphere absolute (ATA), which are minimum requirements as HBOT, in a monoplace chamber (BARA-MED standard, BARA·MED, ETC Biomedical Systems, Southampton, PA, USA), five sessions per week. All patients had secretory otitis media caused by HBOT and required placement of tympanostomy tubes. During hospitalization, compression therapy and aggressive ankle range-of-motion exercise/calf muscle training were continued. All VLUs healed, namely, epithelization was confirmed in 100% of the wound area, after 17–66 sessions of HBOT, of which four of nine cases required more than 30 sessions. No correlation between VLU size and the number of HBOT sessions required was found. **Figure 1** shows a representative case.

## Discussion

The mechanism of action of HBOT on VLU remains unclear; however, HBOT is known to accelerate angiogenesis and collagen synthesis in chronic wounds by correcting improper oxygen delivery.^6)^ The evidence supporting the efficacy of HBOT for the treatment of VLU is also scarce. Two recent prospective trials failed to demonstrate the superiority of HBOT in terms of healing rate when compared with placebo or standard treatment.^7,8)^ These trials included VLUs that responded poorly to a 4 week standard treatment. By contrast, Bass reported that 15 of 19 VLUs, which had not healed for a median of 10.1 years, healed after a median of 60.7 (10–200) h of HBOT.^9)^

Considering that the majority of VLUs heal by compression therapy±superficial venous surgery, most VLUs heal when venous hypertension is controlled even if the course is slow. Conversely, persistently nonhealing VLUs despite standard treatment is mostly due to the seriously impaired systemic/local healing ability of the patients. Indeed, changes in fibroblast and macrophage phenotypes caused by chronic inflammation and iron overload are related to tissue destruction in VLUs, and this condition can persist after the removal of venous hypertension.^10)^ This justifies the use of adjunctive therapy to aid the healing of the wound and may also explain why no correlation was found between VLU sizes and the number of HBOT sessions required. Accordingly, the application of HBOT may need to be limited to treatment-resistant VLUs to elucidate the efficacy of HBOT, as shown in this report and the report by Bass.^9)^

HBOT is indicated for various acute (gas embolism, carbon monoxide poisoning, anaerobic bacterial infection, etc.) or chronic (refractory ulcers due to peripheral circulatory failure, osteomyelitis, radiation injury, etc.) conditions. Oxygen poisoning in the brain/lung and pulmonary overinflation syndrome are known as major complications caused by HBOT, but these are rare as long as the system is properly manipulated. Conversely, barotrauma particularly in the middle/inner ear is frequently encountered. Since 2018, the cost for HBOT is ￥30,000 per day and the number of acceptable HBOT sessions per admission covered by National Health Insurance in Japan has been limited to 30, which was ￥2,000 per day, and the number was unlimited previously. The change was mainly due to cost-effectiveness; however, the current results suggest that more sessions would be required for the majority of VLUs to heal. The best HBOT protocol for VLU has not been elucidated. Longobardi et al. reported a reduced healing rate when 2.4 ATA, 66 min, twice a day were used, which is higher pressure and more frequent use of HBOT compared with those of our protocol.^8)^ This result was possibly related to too much oxygen toxicity. Further pieces of evidence are necessary to elucidate the selection of appropriate candidates and modes of HBOT in future studies.

Although we focused on HBOT in the current report, each adjunctive treatment has advantages and disadvantages. Hence, the indication of each treatment or combination must be decided on the basis of wound type, patient’s condition and preference, and available devices in each clinic.

## Conclusion

HBOT could be considered as one of the effective adjunctive therapies to manage treatment-resistant VLUs.
